# Supplementary material for: Designing meaningful continuous representations of T cell receptor sequences with deep generative models
Source: Nat Commun. 2024 May 20;15:4271. doi: 10.1038/s41467-024-48198-0 (PMC11106309; doi:10.1038/s41467-024-48198-0)
Supplement: Supplementary file 1 — Supplementary Information [file 41467_2024_48198_MOESM1_ESM.pdf]

Supplementary Information

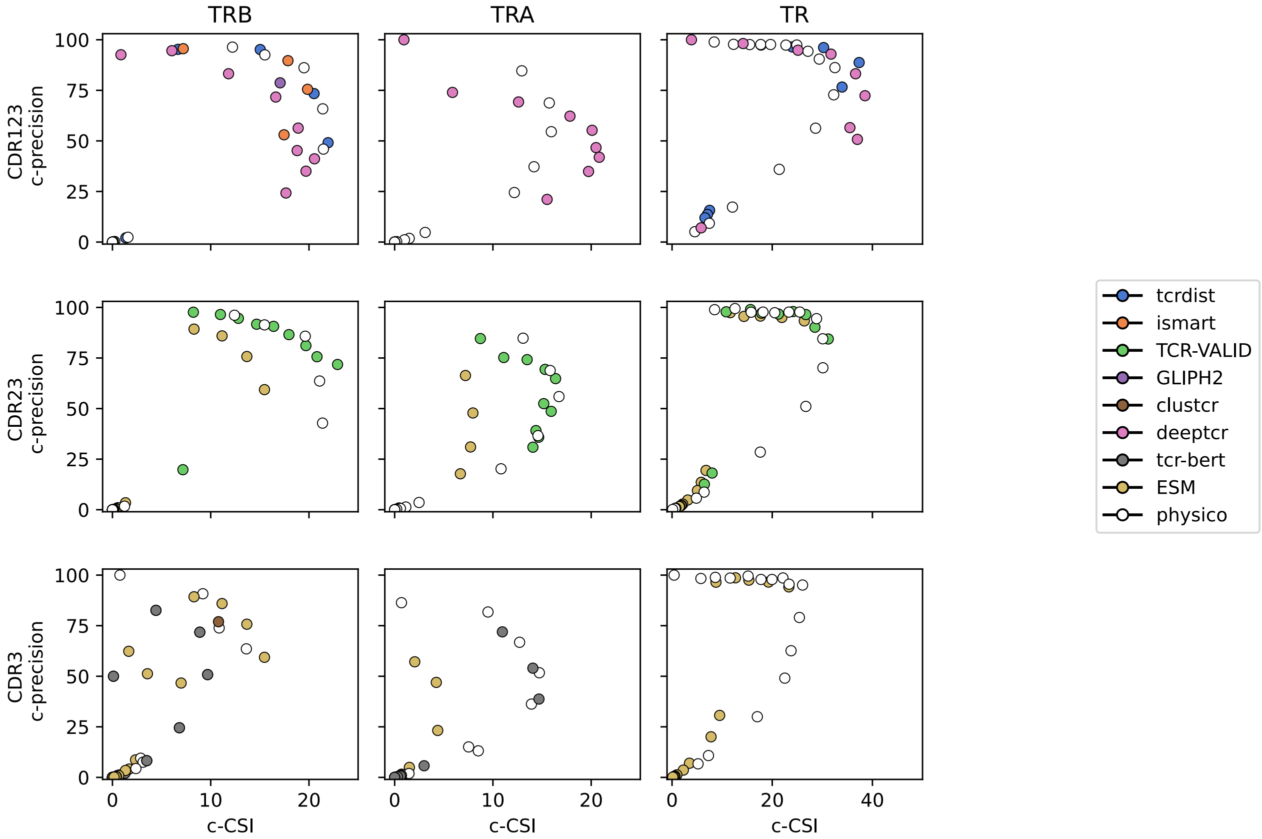

Supplementary Figure 1. Benchmarking TCR-Antigen clustering algorithms Clustering precision versus Clustering-Critical Success Index for TCR-VALID versus both sequence-based (tcrdist and ismart), transformer-based models (tcr-bert and ESM). We additionally benchmarked clustering of TCRs based solely on physicochemically featurized sequences (physico) as those are the base features provided to TCR-VALID. Clustering is based on minsize=3 for all methods. The columns benchmark single chain and paired chains (TRB, TRA, TRB+TRB respectively) whilst the rows benchmark the CDR regions employed(CDR123, CDR23, CDR3 respectively)).

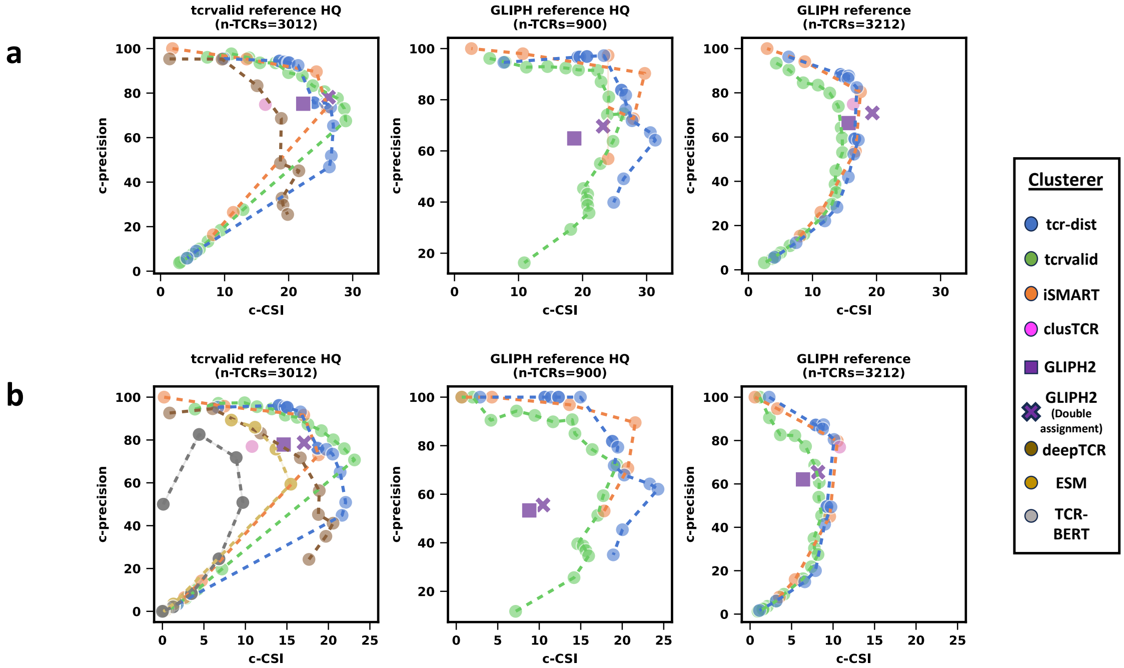

Supplementary Figure 2. Comparing the effect of TCR-antigen reference quality and minimum cluster size on clustering tool performance on TRB chains as evaluated by clustering precision versus clustering Critical Success Index. From left to right, tcrvalid reference of quality >0 as determined by VDJDB, GLIPH2 reference filtered with quality >0 as determined by VDJDB and original GLIPH2 reference [40]. GLIPH2 cluster scoring is undertaken using original webtool output (crosses) and corrected for TCR double assignments (squares) (a) For cluster minsize 2 and (b) minsize 3.

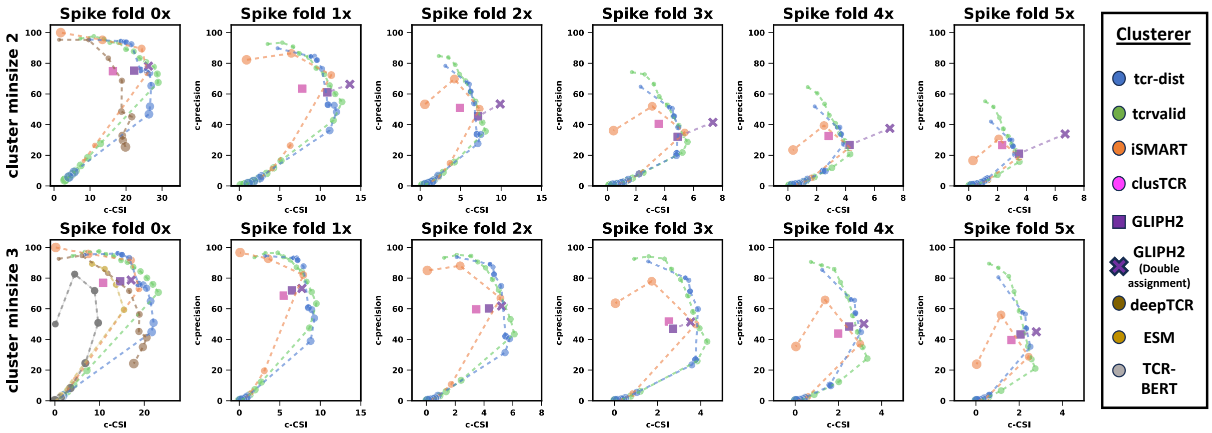

Supplementary Figure 3. Effect of irrelevant TCR spike-in to tcrvalid TCR-antigen reference dataset on TRB chains for spike in folds ranging from 0 to 5x (left to right). Irrelevant TCRs for spike in are obtained as in GLIPH2 [40] publication by sampling the same reference set of CD4s. Benchmarking is undertaken with cluster minsizes 2 (top row) and 3 (bottom row). GLIPH2 cluster scoring is undertaken using original webtool output (crosses) and corrected for TCR double assignments (squares)

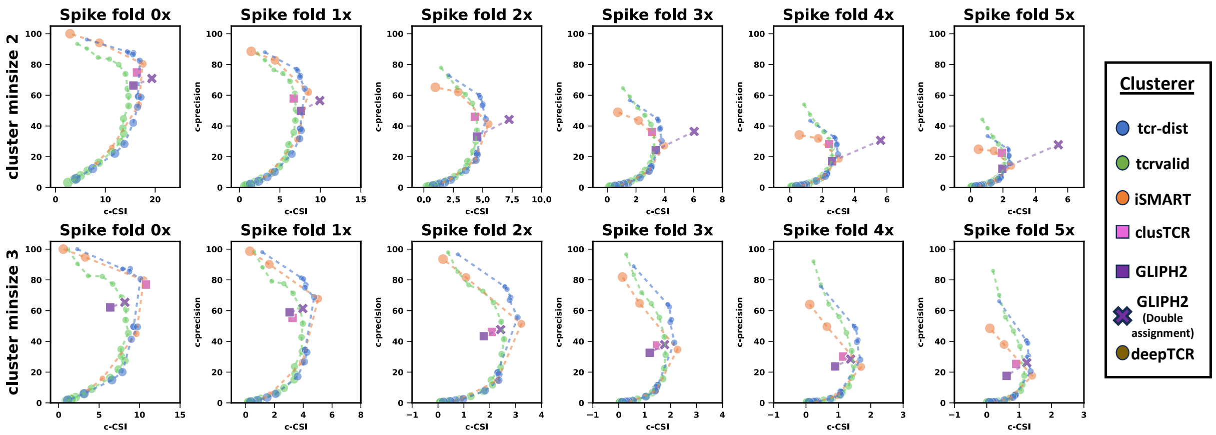

Supplementary Figure 4. Effect of irrelevant TCR spike-in to GLIPH2 [40] TCR-antigen reference dataset on TRB chains for spike in folds ranging from 0 to 5x (left to right). Irrelevant TCRs for spike in are obtained as in GLIPH2 publication by sampling the same reference set of CD4s. Benchmarking is undertaken with cluster minsizes 2 (top row) and 3 (bottom row). GLIPH2 cluster scoring is undertaken using original webtool output (crosses) and corrected for TCR double assignments (squares)

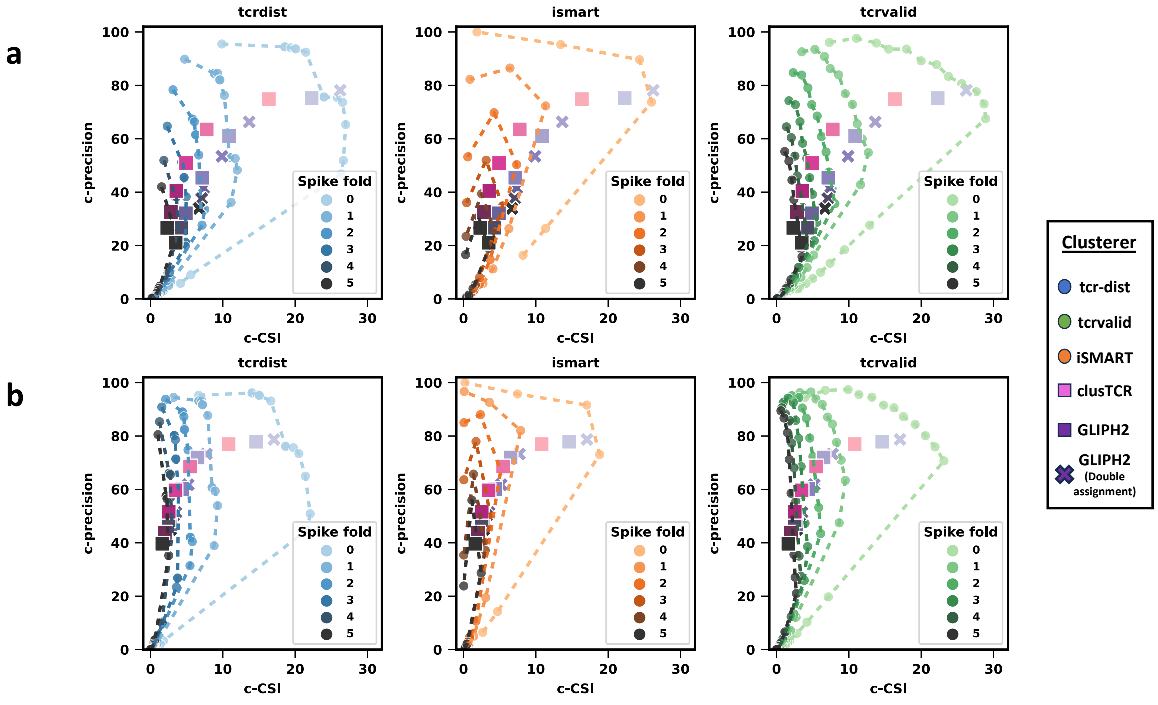

Supplementary Figure 5. Decay of clustering efficiency as increase in folds of irrelevant TCR spike-in to tcrvalid TCR-antigen reference dataset on TRB chains. Benchmarking undertaken for spike in folds ranging from 0 to 5x (light to dark shade) for tcrdist (left, blue), ismart(middle, orange) and tcrvalid (right, green) in combination with GLIPH2 cluster scoring undertaken using original webtool output (crosses) and corrected for TCR double assignments (squares), clustcr. Benchmarking is undertaken with cluster minsizes 2 (a) and 3 (b).

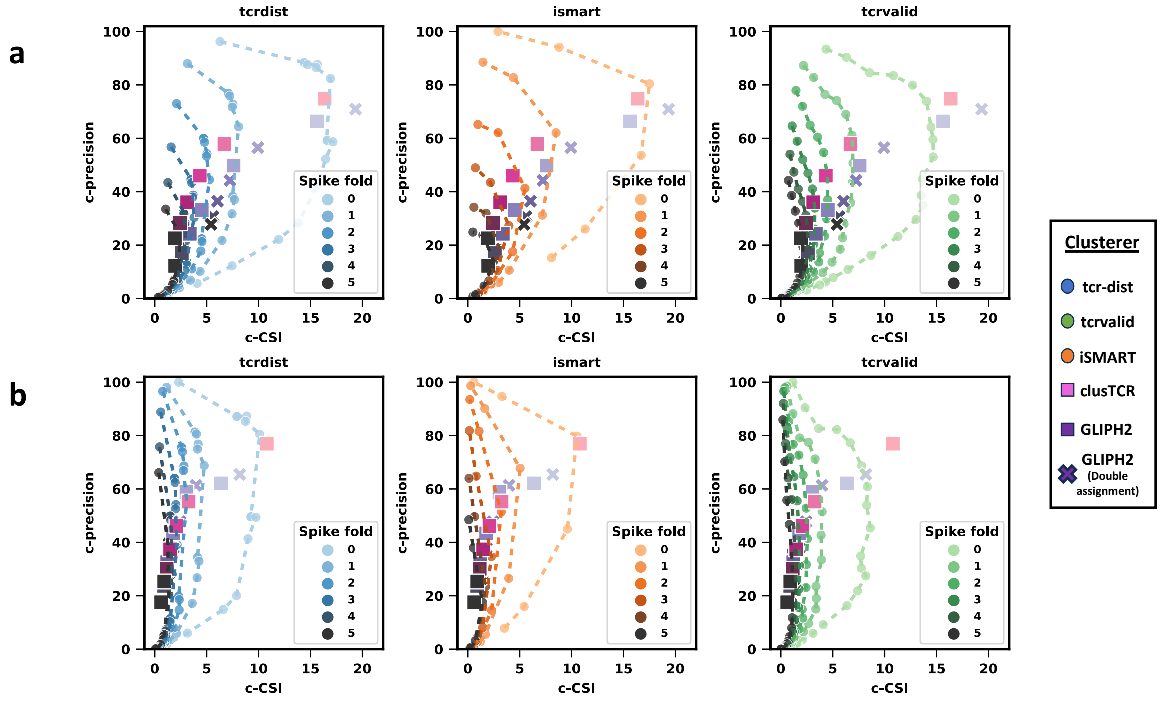

Supplementary Figure 6. Decay of clustering efficiency as increase in folds of irrelevant TCR spike-in to GLIPH2 TCR-antigen reference dataset [40] on TRB chains. Benchmarking undertaken for spike in folds ranging from 0 to 5x (light to dark shade) for tcrdist (left, blue), ismart(middle, orange) and tcrvalid (right, green) in combination with GLIPH2 cluster scoring undertaken using original webtool output (crosses), corrected for TCR double assignments (squares) and clustcr. Benchmarking is undertaken with cluster minsizes 2 (a) and 3 (b).

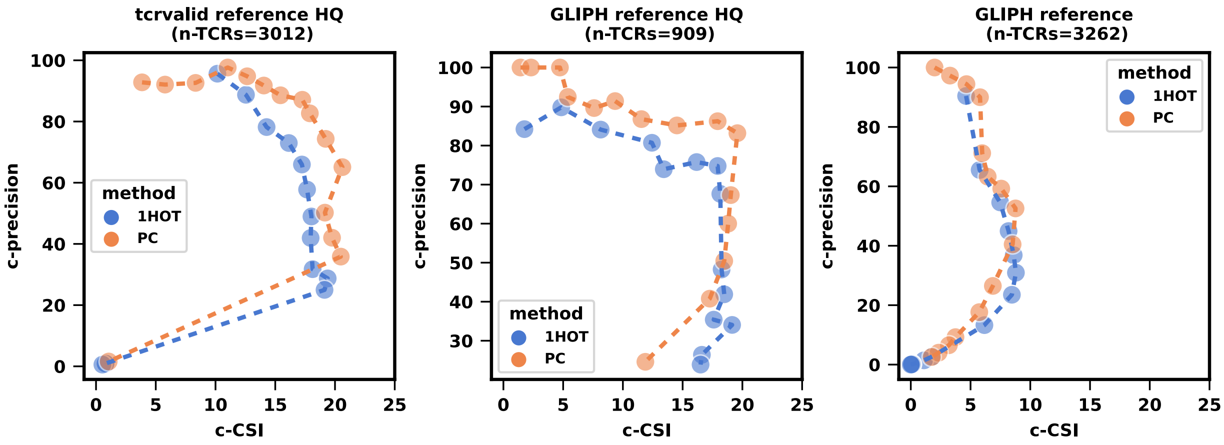

Supplementary Figure 7: Investigation of the difference of One Hot (1HOT) and Physicochemical (PC) TCR sequence (TRB chains) featurizations have on their clustering performance on the tcrvalid high quality reference as defined by VDJDB, GLIPH2 TCR-antigen reference data set [40] in both its original form and filtered for high quality TCRs. Featurized TCR sequences are reduced to 16D using PCA before being clustered using DBSCAN and scored on clustering precision vs clustering Critical Success Index.

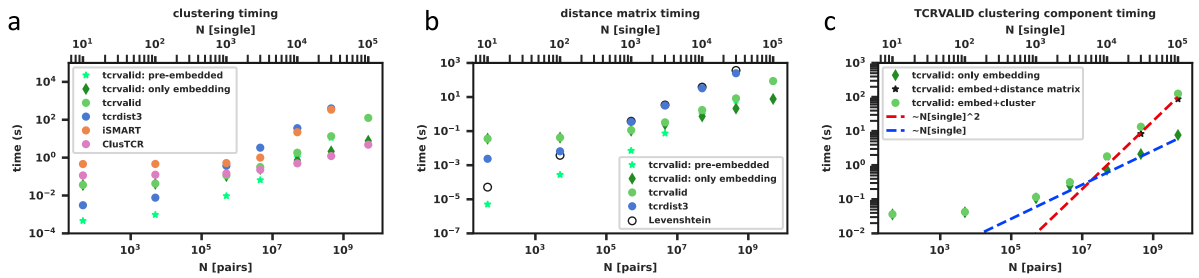

Supplementary Figure 8: Timings of TCR-Clustering and distance matrix calculations inlcuding TCR-VALID separated by components (embedding and clustering). a) Timing of clustering for different methods including TCR-VALID's two main stages for clustering: embedding (tcrvalid: only embedding) and only the clustering with pre-embedded TCRs (tcrvalid: pre-embedded). TCR-VALID embedding scales similarly to clusTCR. b) Timing of distance matrix calculation, compared wth tcr-dist3 and Levenschtein distances, iSMART cannot be compared fairly as it does not compute all pair wise distances. c) Timing of TCR-VALID embedding vs embedding with clustering of full distance calculations, with linear and quadratic relations to guide the eye. TCR-VALID clustering algorithm is the major bottleneck to improving the time scaling of the algorithm further.
