# Supplementary material for: Designing meaningful continuous representations of T cell receptor sequences with deep generative models
Source: Nat Commun. 2024 May 20;15:4271. doi: 10.1038/s41467-024-48198-0 (PMC11106309; doi:10.1038/s41467-024-48198-0)
Supplement: Supplementary file 3 — Reporting Summary [file 41467_2024_48198_MOESM3_ESM.pdf]

nature portfolio

Corresponding author(s): Peter G. Hawkins
Last updated by author(s): 2024-04-5

Reporting Summary

Nature Portfolio wishes to improve the reproducibility of the work that we publish. This form provides structure for consistency and transparency in reporting. For further information on Nature Portfolio policies, see our Editorial Policies and the Editorial Policy Checklist.

Statistics

For all statistical analyses, confirm that the following items are present in the figure legend, table legend, main text, or Methods section.

n/a	Confirmed	
[ ]	[x]	The exact sample size (*n*) for each experimental group/condition, given as a discrete number and unit of measurement
[ ]	[x]	A statement on whether measurements were taken from distinct samples or whether the same sample was measured repeatedly
[ ]	[x]	The statistical test(s) used AND whether they are one- or two-sided<br>*Only common tests should be described solely by name; describe more complex techniques in the Methods section.*
[x]	[ ]	A description of all covariates tested
[x]	[ ]	A description of any assumptions or corrections, such as tests of normality and adjustment for multiple comparisons
[ ]	[x]	A full description of the statistical parameters including central tendency (e.g. means) or other basic estimates (e.g. regression coefficient) AND variation (e.g. standard deviation) or associated estimates of uncertainty (e.g. confidence intervals)
[ ]	[x]	For null hypothesis testing, the test statistic (e.g. *F*, *t*, *r*) with confidence intervals, effect sizes, degrees of freedom and *P* value noted<br>*Give P values as exact values whenever suitable.*
[x]	[ ]	For Bayesian analysis, information on the choice of priors and Markov chain Monte Carlo settings
[x]	[ ]	For hierarchical and complex designs, identification of the appropriate level for tests and full reporting of outcomes
[x]	[ ]	Estimates of effect sizes (e.g. Cohen's *d*, Pearson's *r*), indicating how they were calculated

Our web collection on statistics for biologists contains articles on many of the points above.

Software and code

Policy information about availability of computer code

Data collection	We collected TCR data using the following web resources:<br>iReceptor Gateway (https://gateway.ireceptor.org/login)<br>VDJServer (https://www.vdjserver.org)<br>Zhang et al https://doi.org/10.1126/sciadv.abf5835 via https://github.com/regeneron-mpds/TCRAI/tree/main/data<br>VDJdb (https://vdjdb.cdr3.net)<br>Huang et al., Nature biotechnology 38, 1194 (2020), Supplementary material
Data analysis	Packages required to perform analyses in the manuscript are detailed in full in our github repository (https://github.com/peterghawkins-regn/tcrvalid). For data preprocessing, as described in detail in methods under 'data preparation', we used anarci with the version with git commit c2fd0f7, and spark with version 3.2.1. A minimal set of package requirements to run tcrvalid code is:<br>'matplotlib==3.4.2',<br>'mlflow-skinny==1.24.0',<br>'numba==0.55.1',<br>'pandas==1.2.4',<br>'scikit-learn==0.24.1',<br>'scipy==1.6.2',<br>'seaborn==0.11.1',<br>'tensorflow==2.8.0',<br>'protobuf==3.17.2',<br>'biopython==1.76',

'weblogo==3.7.12',
'datasets==2.7.1',
'Pillow==8.2.0',
'umap-learn==0.5.3'

For manuscripts utilizing custom algorithms or software that are central to the research but not yet described in published literature, software must be made available to editors and reviewers. We strongly encourage code deposition in a community repository (e.g. GitHub). See the Nature Portfolio guidelines for submitting code & software for further information.

Data

Policy information about availability of data

All manuscripts must include a data availability statement. This statement should provide the following information, where applicable:

- Accession codes, unique identifiers, or web links for publicly available datasets
- A description of any restrictions on data availability
- For clinical datasets or third party data, please ensure that the statement adheres to our policy

Collected repertoire level TCR data from the iReceptor Gateway (https://gateway.ireceptor.org/login) and VDJServer (https://www.vdjserver.org) and is publicly available. A list of the repertoire id's of the repertoires used in this study are included in our github (https://github.com/peterghawkins-regn/tcrvalid). Collected paired-chain TCRs with known cognate antigens from two sources; those associated with (https://doi.org/10.1126/sciadv.abf5835, https://github.com/regeneron-mpds/TCRAI/tree/main/data) and VDJdb (all human paired-chain TCRs with a quality 'score' of at least 1 (accessed October 2021) https://vdjdb.cdr3.net) are also publicly available, the dataset has been deposited in our Github repository. We additionally collected TCR-antigen reference data, and CD4 spike in TCRs, from (Huang et al., Nature biotechnology 38, 1194 (2020), https://doi.org/10.1038/s41587-020-0505-4), the dataset and spike-in splits used are available in our Github repository.

Research involving human participants, their data, or biological material

Policy information about studies with human participants or human data. See also policy information about sex, gender (identity/presentation), and sexual orientation and race, ethnicity and racism.

Reporting on sex and gender	ex information of subjects in data collected from public sources may be available in the original data sources from which collected TCR sequences were collected. Sex information is not included in our analysis.
Reporting on race, ethnicity, or other socially relevant groupings	Race and ethnicity information of subjects in data collected from public sources may be available in the original data sources from which collected TCR sequences were collected. Race and ethnicity information was not included in our analysis.
Population characteristics	TCR data collected from public sources are from a wide range of populations.
Recruitment	No recruitment was performed in this study.
Ethics oversight	No recruitment was performed in this study.

Note that full information on the approval of the study protocol must also be provided in the manuscript.

Field-specific reporting

Please select the one below that is the best fit for your research. If you are not sure, read the appropriate sections before making your selection.

☒ Life sciences ☐ Behavioural & social sciences ☐ Ecological, evolutionary & environmental sciences

For a reference copy of the document with all sections, see nature.com/documents/nr-reporting-summary-flat.pdf

Life sciences study design

All studies must disclose on these points even when the disclosure is negative.

Sample size	No formal sample size calculation was performed for this study. A large volume of data was collected to train machine learning models. Models were trained with different sizes of the training dataset. The behavior of the model for smaller training sizes was broadly recapitulated when training with the larger training dataset. This dataset proved sufficient to match the clustering performance of existing clustering tools. The clustering and classification test dataset was collected from public data sources limited by the number of such available paired chain TCR sequences with cognate antigen binding information.
Data exclusions	TCR data without antigen labels were filtered for sequence quality as described in detail in the methods section under 'data preparation'. For the test dataset of antigen labeled TCRs, we collected only paired chain TCRs with a score of at least 1 in the VDJdb to ensure high quality interactions only were present in the dataset. For the classification task, to ensure no very small classes, we retained only TCRs with binding to antigens for which at least 100 unique TCRs were present in the dataset. For the clustering task we only keep TCRs that bind antigens with at least 3 TCRs in the dataset, keep TCRs with length of less than 28 residues (CDR3,CDR3+2) or 35 (CDR3+2+1). These limits were necessary as the CNN model must be trained on fixed length sequences, for which we chose (CDR3+2) of 28 residues which covered the majority of the unlabeled TCR database.
Replication	For trained neural networks all code to reproduce the findings relating to the models latent space behaviors, clustering and classification

	performance are available in our open source code base and have been been confirmed to reproduce the findings of the figures in the manuscript. There may be minor discrepancies for classifiers and UMAP figures owing to differing random seeds.
Randomization	No experimental groups to randomize.
Blinding	Train, validation and test sets were split prior to model training. For clustering and classification tasks, no antigen information had been seen by the unsupervised C-beta-VAE models. For classification performance analysis, cross-validation was performed.

Reporting for specific materials, systems and methods

We require information from authors about some types of materials, experimental systems and methods used in many studies. Here, indicate whether each material, system or method listed is relevant to your study. If you are not sure if a list item applies to your research, read the appropriate section before selecting a response.

Materials & experimental systems

n/a	Involved in the study	
☒	☐	Antibodies
☒	☐	Eukaryotic cell lines
☒	☐	Palaeontology and archaeology
☒	☐	Animals and other organisms
☒	☐	Clinical data
☒	☐	Dual use research of concern
☒	☐	Plants

Methods

n/a	Involved in the study	
☒	☐	ChIP-seq
☒	☐	Flow cytometry
☒	☐	MRI-based neuroimaging

Plants

Seed stocks	*Report on the source of all seed stocks or other plant material used. If applicable, state the seed stock centre and catalogue number. If plant specimens were collected from the field, describe the collection location, date and sampling procedures.*
Novel plant genotypes	*Describe the methods by which all novel plant genotypes were produced. This includes those generated by transgenic approaches, gene editing, chemical/radiation-based mutagenesis and hybridization. For transgenic lines, describe the transformation method, the number of independent lines analyzed and the generation upon which experiments were performed. For gene-edited lines, describe the editor used, the endogenous sequence targeted for editing, the targeting guide RNA sequence (if applicable) and how the editor was applied.*
Authentication	*Describe any authentication procedures for each seed stock used or novel genotype generated. Describe any experiments used to assess the effect of a mutation and, where applicable, how potential secondary effects (e.g. second site T-DNA insertions, mosiacism, off-target gene editing) were examined.*
